# Impact of implantable cardioverter defibrillator on survival in patients with non‐ischemic dilated cardiomyopathy

**DOI:** 10.1002/clc.24032

**Published:** 2023-06-12

**Authors:** Naoya Kataoka, Teruhiko Imamura

**Affiliations:** ^1^ Second Department of Internal Medicine University of Toyama Toyama Japan

To the Editor,

The prognostic impact of implantable cardioverter defibrillator (ICD) therapy in patients with nonischemic dilated cardiomyopathy (DCM) has been of great interest. Omran^1^ and colleagues compared clinical outcomes between ICD therapy and medical therapy alone without ICD in patients with nonischemic DCM. In their study, the rate of sudden cardiac death was lower in patients receiving ICD, whereas the overall survival did not differ between the two therapeutic strategies. Several concerns were raised.

The clinical impact of ICD implantation as primary prevention in patients with nonischemic DCM is accumulating.^2^ A pooled analysis consisting of five landmark ICD clinical trials showed that patients with nonischemic cardiomyopathy had a statistically similar risk of life‐threatening ventricular arrhythmic events but had a higher risk of all‐cause mortality compared to those with nonischemic DCM.^3^ Thus, one of the current issues is the prevention of nonarrhythmic death. In their study of nonischemic DCM,^1^ the overall survival was not affected by ICD implantation. Could the authors attempt to evaluate the cause of death other than sudden cardiac death and discuss how to prevent such a death?

Another concern is the protocol of their study. The baseline characteristics of the two groups (i.e., ICD group vs. medication group) were not statistically matched,^1^ and the inclusion/exclusion criteria should be more clearly stated. The clinical impact of ICD therapy is not similar between primary prevention and secondary prevention, which should be more clearly stated. In addition, supra‐ventricular arrhythmia can be a trigger for ventricular tachyarrhythmia.^4^ The prevalence of atrial fibrillation should be reported and compared between the two groups.

Medical therapy is of great importance to improve clinical outcomes in patients with nonischemic DCM. However, up‐titration of beta‐blockers or any other heart rate‐lowering medications is sometimes difficult because of patients’ sinus bradycardia or atrioventricular block. Cardiac implantable electronic devices, including ICDs and cardiac resynchronization therapy, can secure minimum heart rate and assist up‐titration of these medications, facilitating reverse remodeling and improving mortality and morbidity.^5^ ICD therapy was not associated with greater overall survival in their study. Were the authors able to compare the follow‐up medication data between the two groups?

## CONFLICT OF INTEREST STATEMENT

The authors declare no conflicts of interest.

## Data Availability

Data sharing is not applicable to this article as no new data were created or analyzed in this study.

## References

[1] Salehi Omran H , Naghashzadeh F , Irilouzadian R , Dolatshahi S , Hedayati Goudarzi MT , Salehi Omran MT . The impact of implantable cardioverter defibrillator on the prognosis of nonischemic dilated cardiomyopathy patients compared with standard medical treatments. Clin Cardiol . 2023. 10.1002/clc.24022 PMC10270273 37057368

[2] Anantha Narayanan M , Vakil K , Reddy YN , et al. Efficacy of implantable cardioverter‐defibrillator therapy in patients with nonischemic cardiomyopathy. JACC: Clinical Electrophysiology. 2017;3(9):962‐970. 29759721 10.1016/j.jacep.2017.02.006

[3] Narins CR , Aktas MK , Chen AY , et al. Arrhythmic and mortality outcomes among ischemic versus nonischemic cardiomyopathy patients receiving primary ICD therapy. JACC: Clinical Electrophysiology. 2022;8(1):1‐11. 34454875 10.1016/j.jacep.2021.06.020 PMC8792162

[4] Sarrias A , Villuendas R , Bisbal F , et al. From atrial fibrillation to ventricular fibrillation and back. Circulation. 2015;132(21):2035‐2036. 26596979 10.1161/CIRCULATIONAHA.115.018891

[5] Martens P , Verbrugge FH , Nijst P , et al. Feasibility and association of neurohumoral blocker up‐titration after cardiac resynchronization therapy. J Card Failure. 2017;23(8):597‐605. 10.1016/j.cardfail.2017.03.001 28284756

